# Psychogenic nonepileptic seizures in pediatric population: A review

**DOI:** 10.1002/brb3.1406

**Published:** 2019-09-30

**Authors:** Francesca Felicia Operto, Giangennaro Coppola, Roberta Mazza, Grazia Maria Giovanna Pastorino, Stella Campanozzi, Lucia Margari, Michele Roccella, Rosa Marotta, Marco Carotenuto

**Affiliations:** ^1^ Child Neuropsychiatry Unit Department of Medicine, Surgery and Dentistry University of Salerno Salerno Italy; ^2^ Child Neuropsychiatry Unit Department of Basic Medical Sciences, Neuroscience and Sense Organs University of Bari “Aldo Moro” Bari Italy; ^3^ Department of Mental and Physical Health and Preventive Medicine University of Campania “Luigi Vanvitelli” Naples Italy; ^4^ Department of Psychological, Pedagogical and Educational Sciences University of Palermo Palermo Italy; ^5^ Department of Health Sciences University “Magna Graecia” Catanzaro Italy

**Keywords:** children, psychogenic seizures, video EEG

## Abstract

**Introduction:**

Psychogenic nonepileptic seizures (PNES) are observable abrupt paroxysmal changes in behavior or consciousness that resemble epileptic seizures, but without concurrent electroencephalographic abnormalities.

**Methods:**

In this manuscript, we reviewed literature concerning pediatric PNES and focused on those articles published in the last 10 years, in order to try to understand what the state of the art is at the moment, particularly as regards relationship and differential diagnosis with epilepsy.

**Results:**

Psychogenic nonepileptic seizures have been extensively described in literature mainly in adults and less frequently in children. Despite the potential negative impact of a misdiagnosis (unnecessary investigations and antiepileptic drugs, structured pathological behavioral patterns), in literature there is little information regarding the real prevalence, clinical features, treatment, and outcome of PNES in children and adolescents.

**Conclusion:**

Psychogenic nonepileptic seizures are common but frequently missed entity in pediatric population. Diagnosis could be difficult, especially in those children who have both epileptic and nonepileptic seizures; video EEG and home video can help clinicians in diagnosis. More studies are needed to better classify PNES in children and facilitate diagnosis and treatment.

## Graphical abstract

Psychogenic nonepileptic seizures (PNES) are common but frequently missed entity in pediatric population. A substantial proportion is misdiagnosed as epilepsy and starts AEDs. The diagnosis is more difficult in case of concomitant epileptic seizures. Long term video‐EEG monitoring is useful for an accurate diagnosis. More studies are needed to better classify PNES in children.

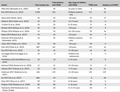

## 1 INTRODUCTION

Psychogenic nonepileptic seizures (PNES) are observable abrupt paroxysmal changes in behavior or consciousness that resemble epileptic seizures but without concurrent electroencephalographic abnormalities (Madaan et al., 2018).

Several terminologies have been used in literature to describe these paroxysmal events, including pseudoseizures, psychogenic seizures, hysterical epilepsy, pseudoepileptic seizures, and nonphysiologic or functional seizures; nevertheless, the term “psychogenic nonepileptic seizures” is preferable as it clearly indicates the nonepileptic nature of these clinical conditions, which are thought to have a psychogenic basis.

Psychogenic nonepileptic seizures have been described extensively in literature mainly in adults and less frequently in children (Patel, Scott, Dunn, & Garg, 2007). There are few population‐based data on pediatric PNES (Madaan et al., 2018); so, it is difficult to accurately estimate their exact incidence or prevalence in children and adolescents (Reilly, Menlove, Fenton, & Das, 2013). Prevalence has been estimated to be 2–33/100,000 (Benbadis & Allen, 2000), and it is presumed to be lower in children than in adults (Madaan et al., 2018). According to Dhiman et al and earlier studies, the prevalence of PNES is 3.5%–20% of children and adolescents undergoing video EEG (vEEG) monitoring (Dhiman, Sinha, & Rawat, 2014; Kutluay, Selwa, Minecan, Edwards, & Beydoun, 2010; Szabó, Siegler, & Zubek, 2012).

Recognition of PNES is important as children could undergo unnecessary investigations and take unnecessary antiepileptic drugs. On the other hand, the early diagnosis and intervention are critical to prevent the behavioral patterns of PNES becoming fully incorporating into the patients' personality and way of life (Verrotti et al., 2009).

Despite the potential negative impact of a misdiagnosis, in literature there is little information regarding the real prevalence, clinical features, treatment, and outcome of PNES in children and adolescents. In this manuscript, we reviewed literature concerning PNES. We have limited our research to articles published in the last 10 years and having children and adolescents as study sample. Given the limited number of articles present, we have included, in our research, even those articles in which the authors considered larger populations, represented by subjects both in pediatric age and in adulthood. We especially focused on those article that better described semiology and clinical features of PNES in pediatric population, in order to try to understand what the state of the art is at the moment, particularly as regards relationship and differential diagnosis with epilepsy.

## 2 RECENT STUDIES ON PEDIATRIC PNES: REVIEW OF THE LITERATURE

Psychogenic nonepileptic seizures have been extensively described in literature mainly in adults (Szabó et al., 2012), but only few studies have tried to semiologically classify psychogenic seizures (An, Wu, Yan, Mu, & Zhou, 2010; Gröppel, Kapitany, & Baumgartner, 2000; Hubsch et al., 2011; Seneviratne, Reutens, & D'Souza, 2010; Wadwekar, Nair, Murgai, Thirunavukkarasu, & Thazhath, 2014). The literature about PNES in children is much more limited (Dhiman et al., 2014).

Reilly et al. (2013), in their article of 2013, reviewed literature investigating all studies on pediatric PNES until that time. According to the authors, the study by Szabó et al. (2012) in 2012 was most comprehensive, involving analysis of 75 events in 27 children among a total sample of 568 patients who underwent video‐EEG monitoring; among them, patients with PNES were 27, 18 with PNES only and nine with epilepsy and PNES.

In turn, in 2012, Szabò et al had identified a previous study by Patel et al in 2007, as the only one assessing semiology of PNES in childhood until that moment. Authors monitored 68 patients with a clinical diagnosis of nonepileptic seizures; patients had been divided into two groups (less than 13 years and 13 years and older); among them, 59 patients had at least one event during the video EEG; so, authors included only them in their study with the aim to compare clinical features in children younger than 13 years and the adolescent group (Patel et al., 2007; Szabó et al., 2012).

In Table 1, we have collected recent studies available in the pediatric age group assessing PNES in childhood, after Szabò et al. (Table 1).

**Table 1:** Recent studies on pediatric psychogenic nonepileptic seizures (PNES)

Author	Total sample size	Total patients with PNES	Age range of children with PNES	PNES only	Epilepsy and PNES
Pillai 2012 (Pillai & haut, 2012)	39	39	16 years or older	0	39
Kim 2012 (Kim et al., 2012)	1,108	143	Pediatric patients only	111	32
Alessi 2013 (Alessi, Vincentiis, Rzezak & Valente, 2013)	42	42	<18 years	25	17
Akdemir 2013 (Akdemir, Uzun, Özsungur & T opçu, 2013)	34	34	12–17 years	34	0
Yi 2014 (Yi et al., 2014)	25	25	8–19 years	17	8
Dhiman 2014 (Dhiman et al., 2014)	56	56	<18 years	47	9
Plioplys 2014 (Plioplys et al., 2014)	55	55	8.6–18.4 years	39	16
Rawat 2015 (Rawat et al., 2015)	44	44	<16 years	44	0
Sawchuk 2015 (Sawchuk & Buchhalter, 2015)	32	29	Pediatric patients only	22	7
Yadav 2015 (Yadav et al., 2015)	90	90	<18 years	90	0
Park 2015 (Park et al., 2015)	887	141	<18 years	125	16
Say 2015 (Say et al., 2015)	62	62	11–18 years	62	62
Cornaggia 2016 (Cornaggia et al., 2016)	10	8	Children and adolescents	2	0
McWilliams 2016 (McWilliams et al., 2016)	20	20	6–19 years	10	9
					1 unclear
Karterud 2016 (Karterud et al., 2016)	11	11	14–24 years	11	0
Valente 2017 (Valente et al., 2017)	53	53	7–17 years	32	21
Anderson 2017 (Anderson et al., 2017)	246	123	12–69 years	123	123
Ito 2017 (Ito et al., 2017)	886	63	0–17 years	0	68
Doss 2017 (Doss et al., 2017)	90	55	8–18 years	55	0
Madaan 2018 (Madaan et al., 2018)	80	80	6–16 years	80	0
Kozlowska 2018 (Kozlowska et al., 2018a, 2018b)	60	60	8–17, 67 years	60	0

[Correction added on 5 November 2019, after first online publication: in Table 1, all values/data listed for the author ‘McWilliams 2016 (McWilliams et al., 2016)’ have been corrected except for the column ‘PNES only’.]

The studies collected are mostly retrospective case reviews including patients admitted to Epilepsy Center for evaluation, whose video‐EEG monitoring were reviewed by child neurologist. Most of them identify only small samples of patients, among which patients often have both epilepsy and PNES. However, from these studies, some precious information concerning incidence, prevalence, and clinical features of pediatric PNES can be collected.

## 3 CLINICAL FEATURES OF PEDIATRIC PNES

Psychogenic nonepileptic seizures should be suspected when a child or adolescent has frequent attacks despite an appropriate medical management, when seizures have atypical clinical features, EEGs are repeatedly normal and these events are exacerbated by stress or other external events (Patel et al., 2007).

Psychogenic nonepileptic seizures in pediatric population have different clinical features compared with adults; moreover, there are also differences in etiology, clinical presentation, associated factors, treatment, and outcome between children and adolescents (Patel et al., 2007).

In pediatric population, recent studies have identified a mean age of presentation of 10.5 years (Madaan et al., 2018) or older: 12.81 years (Valente, Alessi, & Vincentiis, 2017); 14.19 years (Say, Taşdemir, & İnce, 2015); 12.3 years (Dhiman et al., 2014); 11.6 years (Szabó et al., 2012); and 12.9 years (Patel et al., 2007). All these findings are in contrast with another study by Park et al who found a lower mean age, <6 years, at the time of diagnosis of PNES in 141 patients. According to Vincentiis et al. (2006), studies on pediatric PNEs include a majority of patients older than 10 years. However, Patel et al. (2007) noted that limited information is available on the phenomenology of childhood PNES by developmental stages, especially in infants, in which the differential diagnosis is even more difficult.

A female predominance of PNES is seen in adults (67%–74%; An et al., 2010; Gröppel et al., 2000; Hubsch et al., 2011; Seneviratne et al., 2010), as well as in pediatric population (Anderson, Damianova, Hanekomb, & Lucas, 2017; Dhiman et al., 2014; Say et al., 2015; Valente et al., 2017). More recently, Madaan et al. (2018) found a male predominance (56.2% vs. 43.8%) in their sample of 80 patients with PNES. This is in contrast with other authors who had previously described a decrease in the tendency of female predominance in younger groups of children when compared to adolescents (Patel et al., 2007; Kotagal, Costa, Wyllie, & Wolgamuth, 2002). Conversely, Park, Lee, Lee, Lee, and Lee (2015) observed a high proportion of males with psychogenic nonepileptic seizures in younger patients and an equal distribution between both sexes in older patients. Rawat et al. (2015) found no gender differences, instead.

As regards different semiological types of PNES, in adults the most common one is rhythmic motor (Magaudda et al., 2016; Wadwekar et al., 2014), whereas in pediatric studies results are contrasting. Madaan et al. (2018) found that dialeptic and mixed semiology are prevalent in pediatric population. Conversely, according to Valente et al. (2017), PNES semiology was predominantly major motor, followed by minor motor, dialeptic, and finally auras in a sample of 53 patients. These findings are in agreement with previous studies. Say et al. (2015) had found that tremor was the most prevalent ictal motor sign in the entire sample (62 patients) with atonic falls significantly more prevalent in girls; Dhiman et al also supported the evidence that tremors represent the commonest phenomena also in children, not only in adults. Moreover, authors identified negative emotional signs like weeping, moaning, and screaming as important markers for pediatric PNES (Dhiman et al., 2014). On the contrary, Szabó et al. (2012) had found that dialeptic semiology was the commonest one, followed by “aura”.

Nonepileptic seizures present in both psychiatric and nonpsychiatric conditions. According to DSM‐IV, PNES are interpreted as manifestations of “conversion disorder” (Reuber, 2008). More recently, DSM‐5 has proposed new terminology and criteria, identifying this condition under the name of “Functional Neurological Disorder, with abnormal movements,” to potentially foster the collaboration between psychiatrists and neurologists, which is critical to improve both the understanding and care of this neglected group of patients. However, children with PNES usually also have a significant impairment in functioning and psychopathology (Reilly et al., 2013). Kotagal et al found more psychiatric problems in the adolescent group among patients with PNES (Verrotti et al., 2009). Regarding nonpsychiatric conditions, in children the most common one include inattention/daydreaming, staring, sleep myoclonus, stereotyped movements, hypnic jerks, tonic posturing, parasomnias, and movement disorders (Kim, Kim, & Lim, 2012; Reilly et al., 2013; Verrotti et al., 2009).

Doubtless, there is a strong suspicion or positive evidence for a psychogenic cause in childhood PNES (Bodde et al., 2009). Unlike adults, associated stressors, psychiatric, and also organic co‐morbidities are not well defined in pediatric population and there are no specific tools or questionnaire that could be used for their identification, apart from other nonspecific questionnaires assessing behavioral problems in children and adolescents (Madaan et al., 2018). Literature reveals that anxiety and depression are commonly present as co‐morbidities in adults with PNES (Madaan et al., 2018). In children with PNES, the main associated co‐morbidities have been identified in somato‐psychiatric and adversity component to their illness (Plioplys et al., 2014); depressive disorders, followed by ADHD and anxiety (Yi, Kim, Lee, Cheon, & Kang, 2014); conversion disorder, intellectual disability, specific learning disorders (Doss et al., 2017), depression (Rawat et al., 2015); internalizing behaviors (Sawchuk & Buchhalter, 2015).

In their recent study on 80 children aged 6–16 years, Madaan et al found psychiatric co‐morbidities in 13.8% (adjustment disorders, followed by depression and panic disorder) and medical co‐morbidities in 7.5% (chronic illness, such as bronchial asthma, primary nocturnal enuresis, acute intermittent porphyria, and hemolytic uremic syndrome, could induce stress that predisposes to PNES). According to the Authors, stressors have an important role in the etiology of PNES in children, especially family stressors, school stressors, and problem with self (Madaan et al., 2018).

## 4 PNES AND EPILEPSY

Psychogenic nonepileptic seizures are frequently misdiagnosed in children and adolescents, particularly in patients with epilepsy (Verrotti et al., 2009). A significant minority of children referred because of suspected epileptic seizures may not actually have the condition (Kim et al., 2012; Kotagal et al., 2002; Udall, Alving, Hansen, Kibaek, & Buchholt, 2006).

In PNES, concomitant epilepsy has been reported in 5%–20% of patients with psychogenic seizures (Benbadis, Agrawal, & Tantum, 2001; Gates, 2000; Verrotti et al., 2009). In the outpatient epilepsy clinics, the prevalence of PNES has been estimated to vary from 5% to 33%; this percentage is higher (10%–58%) in patients admitted for the evaluation of refractory seizures in tertiary epilepsy centers, likely due to much pertinent diagnostic facilities available, namely video‐EEG monitoring (Dhiman et al., 2014; Pillai & Haut, 2012).

It has been speculated that epilepsy may contribute to the risk of developing PNES not only through biologic mechanisms but also because the experience or observation of epileptic seizures may provide a model to be learned (Asadi‐Pooya & Sperling, 2015; Reuber, 2009).

Demographic and clinical characteristics of patients with both PNES and coexisting epilepsy are similar to those of patients with only PNES (Asadi‐Pooya & Emami, 2013). Montenegro et al in 2008 and Kim et al in 2012 reported similar features for PNES between children with and without epilepsy (Kim et al., 2012; Montenegro et al., 2008). These findings are in contrast with a more recent study by Ito et al. (2017), in which authors suggested that the PNES seen in children with epilepsy have different characteristics: Myoclonus, stereotypies, paroxysmal ocular deviations, and tonic posturing are the most common PNES in children who also have true epileptic seizures (Ito et al., 2017).

Some clinical features help to distinguish PNES from real epileptic seizures. The former can start abruptly, but usually more gradually than the latter; in PNES, shaking is often asynchronous and asymmetrical; motor activity is characterized by phases of major and minor vigor, whereas in tonic‐clonic epileptic seizures there is a gradual decline in the frequency of limb jerks; pelvic thrusting is more common in PNES than in epileptic seizures and eyes and mouth are much more likely to be closed than in real seizures; moreover, eye opening may be resisted and the pupillary light response is preserved in PNES (Reuber, 2008).

According to Pillai et al, frontal lobe epilepsy is more common than temporal epilepsy in patients with epilepsy and concomitant PNES than in patients with epilepsy only. In fact, authors found that frontal seizures were more commonly noted in patients with epilepsy who had concomitant PNES, during video‐EEG monitoring (Benbadis et al., 2001). More recently, also Madaan et al. (2018) have described five children with epilepsy initially suspected to be PNES: 2/5 had focal epilepsy, and 3/5 had frontal lobe semiology.

Frontal lobe seizures mimicking nonepileptic attacks have been labeled as “chameleons,” along with transient epileptic amnesia, by Smith (2012), as they represent disorders easily in disguise in the large and complex field of epilepsy.

To our knowledge, few studies focused on the possible association between PNES and a specific type of epileptic seizures or epilepsy in children and adolescents who present both the conditions. Criteria for concomitant diagnosis of epilepsy have not been consistent in literature reports. Having interictal spikes on EEG records does not necessarily mean that epilepsy is also present, as interictal epileptiform abnormalities have also been reported in nonepileptic conditions and in healthy people (Asadi‐Pooya & Sperling, 2015).

## 5 CONCLUSIONS

Psychogenic nonepileptic seizures are common but frequently missed entity in pediatric population; a substantial proportion is misdiagnosed as epilepsy and starts AEDs. Diagnosis could be difficult, especially in those children who have both epileptic and nonepileptic seizures. After the availability of video EEG, the diagnosis and differentiation of PNES from true epileptic seizures have become easier. Long‐term video‐EEG monitoring is certainly the only tool to make an accurate diagnosis and differentiate these medical conditions, a matter which has certain importance also from a medico‐legal point of view. Nevertheless, we must stress two issues: the first that not always and not everywhere clinicians have the possibility to perform a video EEG in these patients; and the second that this diagnostic procedure has a cost in time and money to consider and evaluate in each individual case.

Parents who have children with epilepsy tend to observe their children more carefully, and home videos captured by family members using their smartphones might help child neurologists to accurately diagnose those psychogenic events that more easily could be misdiagnosed as true seizures. However, diagnosis through home videos can be simpler in the case of seizures with motor semiology; in the case of episodes of absences, it is more difficult to make a correct diagnosis without a video EEG, instead.

We must reiterate that more studies are needed to better classify PNES in children and facilitate diagnosis and treatment. A systematic and uniform classification of childhood PNES would help in better standardization and is required for an earlier diagnosis and wider usage for easy comparison. Doubtless, diagnosis, and treatment cannot disregard a detailed assessment for the underlying psychological stressors and associated co‐morbidities.

In this manuscript, we have tried to provide a practical guide for clinicians who are faced with the complex problem of PNES in the pediatric age. Our goal was to provide clinicians with some indications, through a review of the present literature, in order to better orientate in terms of diagnosis and above all differential diagnosis, with other neuropsychiatric diseases, first of all epilepsy; this is not always simple, especially in the pediatric age.

## CONFLICT OF INTEREST

None decalred.

## Data Availability

Data sharing is not applicable to this article as no new data were created or analyzed in this study.
